# A retrospective analysis of robot-assisted total hysterectomy by transvaginal natural orifice transluminal endoscopic surgery

**DOI:** 10.1016/j.heliyon.2023.e19207

**Published:** 2023-08-20

**Authors:** Penglin Xu, Zhao Zhao, Yanpeng Tian, Yue Li, Yafen Liu, Mei Ji

**Affiliations:** Department of Gynecology, The First Affiliated Hospital of Zhengzhou University, Zhengzhou, Henan, 450052, PR China

**Keywords:** Hysterectomy, vNOTES, Robot-assisted surgery, Transvaginal single-port laparoscopic surgery, Minimally invasive surgery

## Abstract

**Objective:**

The present study aimed to explore the feasibility and safety of robot-assisted total hysterectomy by transvaginal natural orifice transluminal endoscopic surgery (vNOTES).

**Methods:**

In this study, the clinical data of 37 patients who underwent da Vinci robot-assisted total hysterectomy by vNOTES between September 1, 2019 and March 31, 2022 at the Department of Gynecology, the First Affiliated Hospital of Zhengzhou University, China were retrospectively analyzed. Clinical characteristics, operative postoperative complications, surgical outcomes, and postoperative pain scores were collected and analyzed.

**Results:**

The average age of the patients included in the study was 47.43 ± 4.44 years. The body mass index (BMI) was calculated using the formula BMI = body weight (kg)/height^2^ (m^2^). The average BMI was 23.16 ± 2.72 kg/m^2^. Among the 37 patients, 30 patients underwent total hysterectomy and bilateral salpingectomy, of which 11 patients underwent ovarian cystectomy simultaneously. Among these 11 patients, three had bilateral ovarian cysts and eight had unilateral ovarian cysts, with the largest cyst diameter measuring 8 cm. The remaining seven patients underwent total hysterectomy and bilateral salpingo-oophorectomy. The average operative time was 86.19 ± 17.83 min, and the estimated intraoperative blood loss was 24.46 ± 15.40 mL, with no intraoperative complications reported. The time to the first postoperative exhaust was 18.51 ± 6.63 h, and the average postoperative length of hospital stay was 3.81 ± 1.05 days. The postoperative visual analog scale (VAS) pain scores were 5.30 ± 0.91 at 24 h after surgery, 3.30 ± 0.70 at 36 h after surgery, and 1.14 ± 0.92 at 48 h after surgery. Only one patient experienced a fever exceeding 38.5 °C, which resolved after receiving antibiotic treatment.

**Conclusion:**

The use of the da Vinci robot-assisted total hysterectomy by vNOTES demonstrated safety and offers several advantages. These include reduced surgical trauma, an aesthetic incision, decreased pain, and shorter duration of postoperative exhaust time and hospital stay. These benefits contribute to accelerated postoperative rehabilitation.

## 1 Introduction

Hysterectomy is recognized as the most frequently performed major gynecologic surgical procedure for treating various benign uterine conditions such as uterine prolapse, leiomyoma, severe dysmenorrhea, abnormal uterine bleeding and adenomyosis [1]. There are four types of hysterectomy, namely, abdominal hysterectomy, vaginal hysterectomy, laparoscopic hysterectomy, and robot-assisted laparoscopic hysterectomy [2,3]. The selection of the appropriate surgical approach for a hysterectomy depends on the surgeon's expertise, the patients preference, and the complexity of the patient's condition. Vaginal hysterectomy is generally recommended as the primary option due to its less invasive and faster recovery [4]. However, vaginal hysterectomy has certain limitations, such as limited operating space and inadequate exposure of operative field. With the advancements in laparoscopic technology, a growing body of evidence has confirmed that laparoscopic hysterectomy is associated with lower postoperative complications. Therefore, laparoscopic hysterectomy is recommended as the first-line surgical approach by the American Association of Gynecologic Laparoscopists [[5], [6], [7]]. As minimally invasive procedures continue to evolve rapidly and surgical instruments improve, along with an increased focus on rapid rehabilitation, doctors and patients have higher expectations regarding surgical approaches, surgical outcomes and patients' subjective feelings during the perioperative period.

Natural orifice transluminal endoscopic surgery (NOTES) is an emerging minimally invasive surgery, wherein surgical procedures are performed using the body's natural orifices, such as the mouth, rectum, vagina, or urethra [8,9]. Transvaginal NOTES (vNOTES) combines single-port laparoscopic surgery (SPLS) and transvaginal surgery, offering safer and less invasive techniques while addressing the challenges associated with conventional transvaginal surgery in terms of exposure difficulties. However, conventional vNOTES still has significant limitations. Interference between surgical instruments poses challenges, particularly during laparoscopic suturing of the vaginal stump [10]. The introduction of robot-assisted laparoscopic techniques holds promise for overcoming the inherent limitations of conventional laparoscopic surgery [11,12]. Robot-assisted vNOTES provides enhanced three-dimensional (3D) visualization and a clear operative field. The robotic platform offers improved stability and more flexible wristed instruments, effectively compensating for the inherent limitations of conventional vNOTES [13]. In 2015, robot-assisted vNOTES was first applied to hysterectomy [12]. However, there have been limited reports on the use of robot-assisted vNOTES for total hysterectomy. In 2019, our research team performed the first robot-assisted vNOTES total hysterectomy in China, and no perioperative complications occurred in this patient. Therefore, it was believed that robot-assisted vNOTES hysterectomy might be feasible and safe, presenting an alternative. surgical approach for hysterectomy.

This study involved the collection of clinical data from 37 patients who underwent da Vinci robot-assisted total hysterectomy by vNOTES. Various clinical characteristics of these patients, including operative time, intraoperative blood loss, and others, were analyzed. The primary goal of this study was to evaluate the safety and feasibility of robot assisted vNOTES hysterectomy.

## 2 Methods and materials

### 2.1 Ethical approval

The Ethics Committee of the First Affiliated Hospital of Zhengzhou University (2023-KY-0436-001) approved this study. Written informed consent was obtained from all patients.

### 2.2 Study participants

This single-center retrospective study was conducted by the Department of Obstetrics and Gynecology, The First Affiliated Hospital of Zhengzhou University from September 1, 2019 to March 31, 2022. Thirty-seven patients who underwent robot-assisted vNOTES total hysterectomy were enrolled in this study.

The inclusion criteria for this study were as follows: (1) a history of previous vaginal sexual intercourse and (2) the presence of benign uterine diseases, such as uterine fibroids, adenomyosis, and endometrial atypical hyperplasia confirmed by pathology and high-grade cervical squamous intraepithelial lesions. The exclusion criteria were as follows: (1) a history of pelvic inflammatory disease and gynaecological examination indicating suspicious pelvic adhesions; (2) severe endometriosis; (3) no history of sexual intercourse, pregnancy, vaginal stenosis, and vaginal atresia or agenesis; (4) patients with a significantly enlarged uterus as identified by ultrasonography.

### 2.3 Surgical procedure

**Fig. 1:**
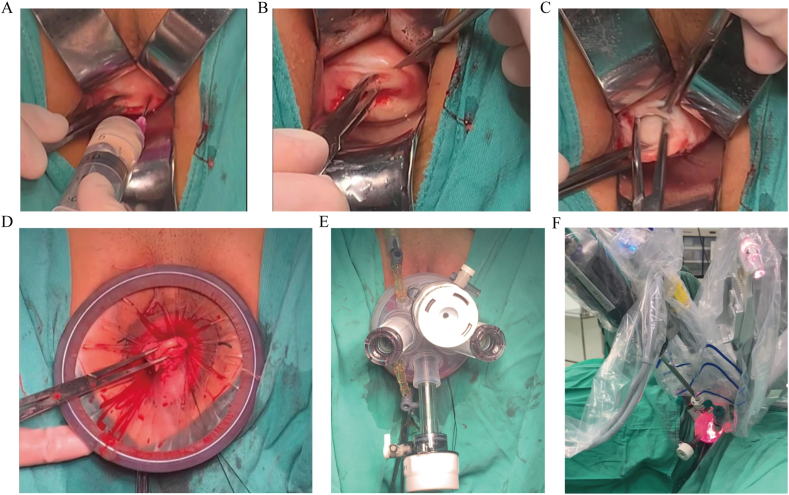
The surgical procedure for robot-assisted vNOTES total hysterectomy. (A) An injecting of 20 mL of diluted epinephrine (1:1000). (B) Circumferential incision of the cervicovaginal junction. (C) Bilateral vesicovaginal interspace and rectovaginal fascia separation via blunt dissection. (D–E) Insertion of a four-hole single port into the vagina. (F) Installation of the robotic arms.

All surgeries were performed by the same experienced gynecologist who had expertise in SPLS and robotic surgery. The robot-assisted vNOTES total hysterectomy procedures were conducted as follows:

All patients underwent standard vaginal and abdominal preparation before the surgery. Throughout the surgery, continuous bladder drainage was ensured using a Foley catheter. Initially, a 20 mL dilute vasopressin solution (1:1000) was injected (Fig. 1A), followed by a circumferential incision at the cervicovaginal junction (Fig. 1B). Blunt dissection was employed to separate the bilateral vesicovaginal interspace and rectovaginal fascia. The left cardinal ligament and uterosacral ligament were exposed and incised, followed by the same procedure for the right cardinal ligament. Circular suturing of the pelvic peritoneum and the surrounding vaginal wall was performed, as illustrated in Fig. 1C and D. A four-hole single port was inserted into the vagina, assembled as illustrated in Fig. 1E and F. The da Vinci Xi Surgical System IS4000 (Intuitive Surgical Sarl, CA, USA) was docked on the patient's right side. The surgeon controlled two robotic instruments through the console, while the assistant surgeon provided support through two ports. Once pneumoperitoneum (10–14 mmHg) was established and maintained, the remaining surgical steps were performed as planned preoperatively. Upon completion of the surgery, the vaginal platform was removed, and the cuff was closed using conventional suturing techniques.

### 2.4 Clinical outcome measures

The clinical outcome measures included operative time, intraoperative blood loss, time to first flatus, duration of urethral Foley catheter retention, length of postoperative hospital stay, and clinical visual analog scale (VAS) pain score (24 h, 36 h, and 48 h after surgery). Wound healing was observed and evaluated 3 months postoperatively.

### 2.5 Statistics

The collected data were analyzed using SPSS version 26.0. Continuous variables with a normal distribution are presented as the mean ± standard deviation, while variables without a normal distribution are presented as medians (interquartile ranges). Enumeration data are presented as percentages. Normally distributed samples were compared using an independent samples *t*-test.

## 3 Results

Thirty-seven patients who underwent robot-assisted vNOTES total hysterectomy were enrolled in this study. The general clinical characteristics of these patients are summarized in Table 1. The average age of the patients was 47.43 ± 4.44 years (ranging from 38 to 55 years). The average body mass index (BMI) was 23.16 ± 2.72 kg/m^2^ (ranging from 18.8 to 28.9 kg/m^2^). Among the 37 patients, 21 had a history of surgery, with 10 patients having undergone cesarean delivery and the remaining patients having a history of abdominal surgery for non-gynecological and non-obstetric conditions. Additionally, seven patients had medical diseases, including three patients with hypertension, one with myocardial ischemia, one with diabetes mellitus and hypertension, and two with diabetes mellitus.

**Table 1:** Clinical features and essential data of patients.

Variables	‾X ± s or n（%）
Age	47.43 ± 4.44 (38–55)
BMI	23.16 ± 2.72 (18.80–28.90)
*Number of deliveries*	1.92 ± 0.80 (1–5)
Vaginal *delivery*	30（81.08）
Cesarean *delivery*	10（27.03）
*History of abdominal surgery*	11（29.73）
Medical *comorbidities*	7（18.92）

All patients successfully underwent the planned surgical procedures without any intraoperative complications. The average overall operative time was 86.19 ± 17.83 min, and the estimated intraoperative blood loss was 24.46 ± 15.40 mL. These results are presented in Table 2. Of all the patients enrolled in this study, 30 underwent robot-assisted vNOTES total hysterectomy and bilateral salpingectomy. Among these patients, 11 underwent concomitant ovarian cystectomy, with three patients having bilateral ovarian cysts and eight having ovarian cysts. The postoperative pathological findings are presented in Table 3. The remaining seven patients underwent hysterectomy and bilateral salpingo-oophorectomy. The average time to the first postoperative exhaust was 18.51 ± 6.63 h (ranging from 9 to 41 h), and the average postoperative length of hospital stay was 3.81 ± 1.05 days (ranging from 3 to 7 days). These results are presented in Table 2. The postoperative VAS pain scores were 5.30 ± 0.91 at 24 h after surgery, 3.30 ± 0.70 at 36 h after surgery, and 1.14 ± 0.92 at 48 h after surgery, as presented in Table 4. Only one patient experienced a fever above 38.5 °C, which resolved with antibiotic treatment. The urethral Foley catheter was successfully removed on postoperative day 2 in all enrolled patients. The clinical diagnoses are presented in Table 5. The primary etiologies for hysterectomy were uterine fibroids and adenomyosis. All clinical diagnoses were consistent with the postoperative pathological diagnoses. All patients were followed up at 3 months after surgery, and satisfactory wound healing of the vaginal stump were observed in all cases.

**Table 2:** Perioperative *Indicators* of patients.

Variables	‾x ± s or n（%）/（range）
Duration of surgery (min, ‾x ± s)	86.19 ± 17.83 (59–150)
Intraoperative bleeding volume (ml, ‾x ± s)	24.46 ± 15.40 (5–50)
Bilateral salpingo-oophorectomy [n, (%))]	7（18.92）
Intraoperative complications [n, (%))]	0（0）
Postoperative complications [n, (%))]	1（2.7）
Fever [n, (%))]	1（2.7）
Intestinal recovery time (h, ‾x ± s)	18.51 ± 6.63 (9–41)
Postoperative hospital stay (d, ‾x ± s)	3.81 ± 1.05 (3–7)

**Table 3:** Pathological results of the ovarian cyst.

Pathological results	Number of cases
*Corpus* luteal *cyst*	4
*Endometriotic* cysts	1
*Follicular* cysts	6

**Table 4:** Postoperative *VAS* scores of Patients.

Case	24 h after surgery	36 h after surgery	48 h after surgery
1	6	4	1
2	5	4	0
3	5	3	1
4	4	3	2
5	6	5	3
6	6	5	0
7	6	3	0
8	7	3	0
9	5	4	1
10	5	3	1
11	5	3	1
12	5	4	3
13	4	3	2
14	4	3	2
15	4	3	2
16	4	2	1
17	5	4	1
18	5	3	1
19	5	4	1
20	5	3	0
21	6	3	0
22	7	2	0
23	6	3	0
24	6	3	0
25	6	2	0
26	5	3	1
27	5	4	1
28	4	3	1
29	4	3	2
30	4	3	2
31	5	3	2
32	6	4	3
33	6	3	1
34	6	3	1
35	6	3	2
36	7	4	2
37	6	4	1
‾x ± s	5.30 ± 0.91 (4–7)	3.30 ± 0.70 (2–5), *P* < 0.05	1.14 ± 0.92 (0–3), *P* < 0.05

**Table 5:** C*linical diagnosis* of 37 patients.

C*linical diagnosis*	No. of cases (No, %)
Uterine leiomyomas	10 (27.03%)
Adenomyosis	11 (29.73%)
Uterine leiomyomas combined with adenomyosis	5 (13.51%)
Uterine leiomyomas/adenomyosis combined with endometrial polyps	2 (5.41%)
Endometrial hyperplasia	1 (2.70%)
Cervical squamous intraepithelial lesion	4 (10.81%)
Uterine leiomyomas/adenomyosis combined with cervical squamous intraepithelial lesion	3 (8.11%)
Uterine leiomyomas/adenomyosis combined with endometrial hyperplasia	1 (2.70%)

## 4 Discussion

Over the past three decades, there have been remarkable advancements in surgical techniques. Traditional open surgery has been rapidly replaced by minimally invasive approaches such as laparoscopy, robot-assisted laparoscopic surgery, SPLS, and transvaginal SPLS [[14], [15], [16]]. Concurrently, perioperative management has gradually shifted from conventional models to the concept of accelerated rehabilitation surgery. These technological advancements and evolving concepts have yield significant improvements in surgical quality, clinical outcomes and health economics. While the American College of Obstetricians and Gynecologists recommends vaginal hysterectomy as the primary choice [17,18], patients and surgeons alike consistently strive for minimally invasive or even non-invasive procedures that prioritize rapid recovery and minimal surgical incisions. Robot-assisted surgery combined with vNOTES appears to be a promising surgical approach that aligns with these goals [9].

Robot-assisted vNOTES had been applied in total hysterectomy and sacrocolpopexy [9,[19], [20], [21]]. Lee et al. first reported four cases of robot-assisted vNOTES hysterectomy in 2015 [12], subsequently drawing increased attention to this approach [22,23]. In our study, robot-assisted vNOTES hysterectomy was successfully performed in 37 patients without any intraoperative complications. After a 3-month follow-up, all patients exhibited well-healed vaginal stump wounds. These findings suggest that robot-assisted vNOTES total hysterectomy is feasible and safe, potentially serving as an alternative surgical approach for hysterectomy. However, it is challenging to definitively declare robot-assisted vNOTES hysterectomy as the optimum surgical procedure. A previous study compared the outcomes of conventional vNOTES hysterectomy with robot-assisted vNOTES hysterectomy and found no significant differences in operative time, blood loss, hospital stay, or mean pain scores between two groups [9]. However, robot-assisted vNOTES hysterectomy exhibited lower rates of intraoperative and postoperative complications. Furthermore, when compared to conventional laparoscopic hysterectomy, vNOTES demonstrated faster procedure times, reduced painl, fewer post-operative complications, and shorter hospital stays [24]. These findings suggest that robot-assisted vNOTES hysterectomy might represent an ideal surgical approach.

In this study, the results highlighted significant advantages of using a robotic surgery surgical approach in vNOTES hysterectomy, particularly in terms of postoperative pain relief. The postoperative VAS pain scores were 5.30 ± 0.91 at 24 h after surgery, 3.30 ± 0.70 at 36 h after surgery, and 1.14 ± 0.92 at 48 h after surger. Notably, 10 out of 37 patients experienced complete pain relief within 48 h. Several factors might contribute to these outcomes: 1) the enhanced 3D visualization provided by the robotic system; 2) the absence of abdominal scarring and trocar-related injuries; 3) the use of flexible robotic arms to minimize surgical trauma; 4) decreased surgeon tremors and fatigue. Previous studies have reported a median surgery duration of 143 (114–181) min for conventional vNOTES and 157 (123–180) min for robot-assisted vNOTES [9]. In our study, the average surgery duration was 86.19 ± 17.83 (59–150) min. This shortened surgical duration and reduced postoperative pain directly contributed to shorter periods of postoperative exhaust and hospitalization, setting the stage for rapid postoperative recovery. Among the patients, one patient experienced a postoperative fever with a maximum body temperature of 38.7 °C, which improved after receiving antibiotics therapy.

Based on our extensive experience, the various key considerations for robot-assisted vNOTES total hysterectomy are as follows: 1) Patient selection is crucial for successful surgery. Patients with intraperitoneal adhesions, especially severe rectouterine depression adhesions, pose challenges in accessing the abdominal cavity. 2) Menopausal women were excluded from the study due to the decreased flexibility of vaginal walls caused by reduced sex hormone levels. Menopausal women have a higher risk of vaginal lacerations during vNOTES. 3) The positioning of the patient, with the head in a low position and the buttocks raised, allows for optimal exposure of the surgical field. 4) Compared with conventional transabdominal incisions in laparoscopic surgery, vNOTES carries an increased risk of postoperative infection. Strict disinfection protocols before surgery and postoperative prophylactic anti-infective measures could effectively reduce postoperative infections and associated complications.

The present study aimed to asses the feasibility and safety of robot-assisted vNOTES hysterectomy. The results suggest that robot-assisted vNOTES hysterectomy might be an ideal surgical approach. However, certain limitations should be acknowledged. This was a single-centre, retrospective study with a small sample size. The findings have not been validated by large-scale clinical data, limiting the clinical implications and generalizability of the study. Furthermore, the high cost associated with the da Vinci robotic-assisted surgical system hinders the widespread adoption of robot-assisted vNOTES due to financial constraints. Although previous studies have reported the potential for robot-assisted hysterectomy as a day-care procedure [25,26]. Its implementation in China is still in the early exploration stage. Currently, patients often prefer to stay at the hospital for several days to facilitate early recovery after surgery. However, efforts are being made to establish day-care procedures as the first-line approach for robot-assisted vNOTES. In the near future, it is hoped that all patients can undergo robot-assisted vNOTES using a day-care procedure.

In our study, all 37 patients underwent robot-assisted vNOTES total hysterectomy successfully. These findings suggest that robot-assisted vNOTES total hysterectomy is a feasible and safe surgical approach that has the potential to be an ideal method for hysterectomy. However, it is important to acknowledge the limitations of our study, including its small sample size, single-centre cohort, and retrospective design. Therefore, further investigation is warranted in a multi-center setting with a larger sample size to validate and generalize these results.

## Funding

This work was supported by 10.13039/501100006407Natural Science Foundation of Henan, China (grant no. 222300420559).

## Ethics approval and consent to participate

The patients provided written informed consent to participate.

## Patient consent for publication

Written informed consent was obtained from the patients for publication of the data and images in this study.

## Author contribution statement

Penglin Xu: Conceived and designed the experiments; Performed the experiments; Analyzed and interpreted the data; Contributed reagents, materials, analysis tools or data; Wrote the paper.

Yanpeng Tian: Analyzed and interpreted the data; Contributed reagents, materials, analysis tools or data; Wrote the paper.

Mei Ji: Conceived and designed the experiments; Performed the experiments.

Zhao Zhao; Yue Li; Yafen Liu: Performed the experiments.

## Data availability statement

Data will be made available on request.

## Additional information

No additional information is available for this paper.

## Declaration of competing interest

The authors declare that they have no known competing financial interests or personal relationships that could have appeared to influence the work reported in this paper.
